# The Life Mission Theory IV. Theory on Child Development

**DOI:** 10.1100/tsw.2003.116

**Published:** 2003-12-11

**Authors:** Soren Ventegodt, Joav Merrick

**Affiliations:** The Quality of Life Research Center, Copenhagen K, Denmark

**Keywords:** quality of life, QOL, philosophy, holding, holistic process theory, life mission theory, human development, parental education, holistic medicine, public health, Denmark

## Abstract

We can identify five important needs that children have: the need for acknowledgment, acceptance, awareness or attention, respect, and care. If these needs are not met, children will modify themselves by denying central parts of their nature in order to adjust to their parents and the situation at large. When a child denies his or her talents, powers, and gender or aspects thereof, he or she loses quality of life, the ability to function, and physical or mental health. The loss of ability takes the form of diminished social ability, psychosexual potency, joy, energy, and fantasy while playing, as well as diminished ability to concentrate, focus, and learn. Many modifications result in a child with severely damaged self-confidence, self-worth, and poor performance. A child more or less deprived of self-worth cannot enjoy, give, or receive. A child deprived of emotions turns cold, rational, asocial, socially stiff, uncomfortable, and in the extreme case... intentionally “evil”. When a child denies his or her own sex, it becomes invisible, uninteresting, and vague or becomes like the opposite sex in behavior and appearance. The general holistic solution to the vast diversity of symptoms in children with low quality of life is to improve the situation for the child and give the child the holding and support he or she needs. It is very important to realize that a negative belief often has survival value to the child as it helps the child to avoid taking responsibility for problems, which really belong to the parents or other adults. Children have a fine capability for spontaneous healing, and seem to enter this process more easily than adults, given sufficient holding. The symptoms of children with poor thriving ability are often difficult to understand, as they are caused by a complex combination of self-modification in five existential dimensions. This often leads to complex medical diagnosis, giving the idea that the child is sick and without therapeutic reach, while sufficient holding could solve the problem. If holding and support of the child is not enough, the situation must be carefully analyzed to find other possible causes of poor quality of life, health, and functional ability. Education of the parent in holding is often mandatory. Most children with bad thriving ability can thus be helped by simple means.

## INTRODUCTION

We can identify five important needs that children have, such as those for acknowledgment, acceptance, awareness or attention, respect, and care[1]. If these needs are not met, children will deny central parts of their nature[2,3] in order to adjust themselves to their parents and the situation.

The way a child can modify him- or herself is manifold. When a child denies his or her talents, powers, and gender or aspects thereof, he or she loses quality of life, the ability to function, and physical or mental health. The loss of ability takes the form of diminished social ability, psychosexual potency, joy, energy, and fantasy while playing, as well as diminished ability to concentrate, focus, and learn. Many modifications result in a child with severely damaged self-confidence, self-worth, and poor performance. A child more or less deprived of self-worth cannot enjoy, give, or receive. A child deprived of emotions turns cold, rational, asocial, socially stiff, uncomfortable, and in the extreme case… intentionally "evil". When a child denies his or her own sex, it becomes invisible, uninteresting, and vague or becomes like the opposite sex in behavior and appearance.

The general holistic solution to the vast diversity of symptoms in children with low quality of life is to improve the situation for the children and give them the holding and support they need. The support, often called "processing" in holistic therapy, must be threefold: to help the children confront difficult emotions and thereby contain their own feelings, to help them understand themselves and their environment, and to help them let go of negative beliefs, experiences, and attitudes[4]. It is very important to realize that a negative belief often has survival value to children as it helps them to avoid taking responsibility for problems, which really belong to the parents or other adults. Children have a fine capability for spontaneous healing, and seem to enter this process more easily than adults; when a child refuses to let go of a negative belief, it is wise to assume that this belief has survival value, and you must start analyzing the environment to find an external reason for the negative attitude. If a child is "pushed" to change his or her attitude in the process, this can often be experienced by the child as scolding and it might lead to more negative self-modifications. In general, therefore, holding is much more important than processing when it comes to children.

With appropriate holding and processing, giving the child the needed qualities of love, caring, and awareness at a later point in time, he or she spontaneously gets into a state of healing[4] and lets go of the modifying decisions and returns to his or her original self, reappearing as intelligent, delightful, interested, loving, playful, and imaginative. As the self-modification of the child is often a very fair reaction to a family situation, it is often necessary to support the parents too, and educate them in holding to accomplish a lasting improvement of the quality of life, health, and functional ability of the child. If holding and support of the child is not enough, the situation for the child must be carefully analyzed around the clock to find other possible causes of poor quality of life, health, and the ability to function.

## LOSS OF HEALTH, COPING, AND QUALITY OF LIFE IN CHILDHOOD

It has been very difficult to understand disturbances of child development and the wide range of abnormalities and symptoms these children present[5]. Many of the problems seem elusive and are difficult to define: lack of normal spontaneous activity, low creativity and fantasy during play, poor social skills, diminished ability to give and take, subnormal curiosity, little joy of life, faint intuitive competence, dumbness, or lack of mental sharpness.

Most parents accept their children as they are, explaining even a very poor quality of life by reference to the child's genes. Sometimes a child's poor quality of life evidently springs from inadequate holding by its parents. But severe developmental disturbances, without any known biological or hereditary etiology, can also been found in children living in seemingly good families, with no obvious signs of violence, neglect, or child abuse[6,7]. These poorly adjusted children often end up as our patients in adult life, and in therapy, often find or rediscover extremely painful, traumatic events experienced in their childhood[8]. Interestingly, their parents seldom realized the subjective dimensions of horror and emotional intensity felt by their children in the traumatic events. Often the emotional pain from these events is so strong that the children found it necessary to modify their personalities to avoid the existential pain.

As the patient integrates the traumas during the therapy, it becomes gradually clearer why these painful shifts in personality were necessary for the survival of the child. When the patient finally takes full responsibility for his or her own emotional pain, he or she regains the lost quality of life, health, and ability to function, making the holistic therapy a worthwhile effort.

The following theory and reflections are based on the case records of about 200 patients who have been through the holistic therapy that has attempted to integrate their traumas from early childhood[9,10]. Half the patients came from average middle-class families. The theory of child development explains why a lack of holding causes the child to feel so much pain as to induce personality modifications in the child.

## THE NEED FOR HOLDING

We may assume that a child is born with certain potentials — to perceive coherently, to walk, to learn a language, and so on. The child is also equipped with a purpose in life in order to bring out these potentials. For this the child depends on its parents for about the first 15 years of life. They must give him/her acknowledgment, attention, respect and emotional contact, bodily care, and acceptance, if the child is to have an uncomplicated and untroubled childhood. These five fundamental needs sum up the child's need for holding. The child needs holding because it does not have the power to realize its purpose of life on its own. At birth, the womb's holding gives way to the parents' care. This is a difficult task, and our work with spontaneously regressive therapy[4] seems to reveal that most children acquire traumas in early childhood because of inadequate holding[10].

As the child develops its body, feelings, mind, and brain, the need for holding becomes gradually less. One day the child can stand on its own, existentially too, if the upbringing has been successful.

In the healthy womb, we receive unconditional love and acknowledgment of the person we are, nutrition, protection, constant physical contact (intensive care), a well-defined space to live in (respect), emotional contact, awareness, and maybe most important, acceptance of our body and gender at the deepest and most fundamental biological level. Although as a fetus we carry strange histocompatibility genes, we are not rejected, but accepted as ourselves.

The child must have all these existential needs continuously fulfilled in order to develop in a joyful and harmonious way, expressing and mastering its many talents one by one, supported by the powers of the parents. When the child has developed mind, body, and feelings of his own, the holding is no longer needed.

## SELF-MODIFICATION IN RESPONSE TO LACK OF HOLDING

Unfortunately, no parents can fulfill all the needs of the child all the time. As soon as the child misses proper holding, the full expression of its purpose in life, its life mission, is jeopardized, and the child finds him- or herself in emotional or existential pain[2]. If the child judges the pain to be unbearable, he or she modifies him- or herself by negative decisions that deny primary or secondary talents. Soon the child has lost some of his or her ability to love; some of the mental, emotional, or physical strength; and some of the natural joy. The child continues to modify him- or herself in a persistent search for acknowledgment, awareness, respect, care, and acceptance.

The child often makes numerous decisions throughout childhood, often surprisingly imaginative. Some of these have negative wordings and some positive wordings, but both the seemingly positive and negative decisions pull the child away from his or her true self, the natural state of being. Depending on the content of the modifying decisions, the child can be overactive or passive, overemotional or overrational, dumb or bright, cheerful or dull, sexually overactive or neutral, opposite-sex–like in behavior and appearance, social or antisocial, loving or hateful, etc. Learning disabilities, behavioral abnormalities, anxiety, and aggression are also part of this picture. If the child denies his own *I* or denies the abstract *you*[11] — any person who is not the *I* — the child may turn autistic. If the child denies reality at large, it may become psychotic. If the child deems the world to be bad and life in general to be impossible, he or she may become depressed. If he or she denies all the physical power, this can even turn into serious illness and maybe even death. A system for the denials and their consequences can be found in Table 1.

## REVERSING THE DESTRUCTIVE MODIFICATIONS OF PERSONALITY IN CHILDREN

It is important to help the child understand that lack of holding forces the child to take negative decisions that destroy his or her genuine personality and core talents. The child has one power intact at all times and that is the power of making decisions to avoid pain and be more fit.

The process of crises and modification is highly reversible. Any person close to the child can help by giving the holding needed. The child has a natural drift towards healing and is just waiting to receive what he or she never had and wanted ever since. So anybody with a heart and the ability to see and acknowledge the child, accept and give awareness, respect, and care can help. And no matter how serious the developmental disturbance, the child can be helped if you can reach out and give what is needed. In the moment when you reach it, all the emotional pain — like hopelessness, anger, sadness, or fear — will surface. Allowing the child to be sad or angry or shy is essential for helping the pain to come out. The negative essence of the gestalts must be confronted as in therapy with adults. But the stronger and more convincing the holding is, the less pain the child will feel.

In many situations, the child will reject the offer of holding from an adult. What seems to be lack of trust is a very clear intuition that it is not good to let go of the defense systems as long as they are still needed in the family.

A much more fruitful approach is to educate the parents of a child with developmental disturbances to give it the five qualities of holding that it needs. This can be done with the parents and the family together in a therapeutic session, where the physician will train the parents in seeing, touching, and meeting the child. Even some physical illness can heal as soon as the parents learn to care for, respect, and see the child. It is often difficult to believe that this little, a few hours of practice in the clinic, does so much for the child.

It is important to notice that you can only give the child what you can give yourself. Education and high levels of formal competence will not help a person help his child, if he does not respect himself, understand himself, accept himself or love himself. So, often the parents must go in therapy to be the parents their children need. The child is very often the thermometer of the family. When the child is happy again, the whole family has been healed.

## TABLE 1

| **1. Existential Dimension** | **2. Holding Needed** | **3. Fail** | **4. Consequence** | **5. Symptom** | **6. Diagnosis** | **7. Therapy as Compensation** |
|---|---|---|---|---|---|---|
| I. Wholeness (soul); core talent, love, I-you relationship | Unconditional love and acknowledgment of core talent/genuine self | No love, conditional love, no acknowledgment | Denial of self, resignation, suppression of talent, loss of motivation, shift to mental position (ego), going for love | Weak I, unloving personality, poor relations, numbness, no interest in other children or adults | Autistic, neurotic, psychopathic, psychotic | Unconditional love and acknowledgment of core talent/genuine self. The child must be seen as the gifted person he or she really is.<br><br>Teaching the parents<br><br>Parents into therapy |
| II. The powers: being, worth, enjoyment, intuition [center of body]<br><br>“Havingness” and respect [solar plexus]<br><br>Expression of self, attention, intelligence, “doingness”, coherence with surrounding world [center of mind] | Attention, respect, care, mental, emotional and physical contact | Neglect, carelessness, dominance, abuse, violence | Mental disease, physical disease, poor social ability | Loss of intelligence, anger, fear, dominance, abusiveness, violence, intended evil | | Awareness, respect, care, well-intentioned mental, emotional, and physical contact, acceptance<br><br>Teaching parents the five dimensions of holding<br><br>Parents into therapy |
| IIa. Mind | Attention in the right quality – neutral, with love, with admiration, etc. | Failed or offended mentally, scolded | Low self-esteem, compromised concentration and learning, inhibited behavior, loss of initiative, loss of intelligence | Unaware, lack of concentration, uninterested, no initiative, low imagination, doing without thinking, failures from doing | Mental disease, intelligence deficit (ID) | Awareness in the right quality – neutral, with love, with admiration, etc. Acknowledgment and acceptance<br><br>Teaching the parents<br><br>Parents into therapy |
| IIb. Feelings | Respect, meeting at the “psychic” border between the two souls, the two minds, and the two bodies | Failed, dominated, abused, punished, frozen out | Emotional coldness, poor emotional intelligence, poor social functioning, missing ability to keep and hold own space and borders, evil intention | See left | Anxiety, emotional disturbances, problems with contact, intelligence deficit (ID) | Respect, meeting at the “psychic” border between the two souls, the two minds, and the two bodies, care, awareness, acceptance<br><br>Teaching the parents<br><br>Parents into therapy |
| IIc. Body | Bodily care, loving touch, physical contact | Failed, violently abused or punished, no care, neglect | Feeling worthless, no enjoyment, poor intuition, bad physical centering clumsiness, passivity | Behavioral disturbances, anxiety, aggression, clumsiness, passivity, no motivation or joy of movement | Poor quality of life, poor health, poor functioning | Bodily care, loving touch, physical contact acceptance<br><br>Teaching the parents<br><br>Parents into therapy |
| III. Gender and sexuality | Acceptance, physical contact with intention of acceptance | Abandonment, scolding, sexual abuse, sexual neglect | Shame, escape from body, loss of interest in the other sex, general weakness, loss of joy, loss of enjoyment, no falling in love, no boy- or girlfriend | Invisibility, difficult to like, difficult to accept, not liking, not accepting, repulsive and ugly, too masculine, too feminine | | Acceptance, physical contact with intention of acceptance<br><br>Teaching the parents<br><br>Parents into therapy |

**TABLE 1 continued**

| 1. Existential Dimension | 2. Holding Needed | 3. Fail | 4. Consequence | 5. Symptom | 6. Diagnosis | 7. Therapy as Compensation |
|---|---|---|---|---|---|---|
| Serious damage in all three dimensions: talent, power, and sex, very destructive parents | | | | | Low quality of life, poor ability to function, poor health, severe disturbances in development ID, DAMP? | Five dimensional holding of the child<br><br>Teaching parents the five dimensions of holding<br><br>Parents into therapy<br><br>Removing child from parents |

The five dimensions of holding are awareness, respect, care, acceptance, and acknowledgment, corresponding to the five existential dimensions: mind, feelings, body, gender, and soul (wholeness) of the child. The developmental consequences of failed holding seriously compromise quality of life, health, and the ability to function due to self-modification by the child in order to survive. The symptoms of the child with poor thriving ability are often difficult to understand, as they are caused by a complex combination of self-modification of the five existential dimensions. This often leads to complex medical diagnoses, giving the idea that the child is sick and without therapeutic reach; sufficient holding often solves the problem.

## A CLINICAL EXAMPLE

The theoretical arguments underlying the proposed framework are to be found elsewhere[1,2,3,4]. The reason for our belief comes from very convincing examples in the clinical practice like the following case story.

> *A girl, aged 8 years, with severe back pain with no organic cause at the first session. The patient had suffered severe pain in her back for two years. X-ray and scanning were completely normal. On examination, very tense muscles were found in her back. A slight pressure on the muscles provoked the well-known pain to reappear. When the patient is "met", getting care and attention (holding), she starts to cry. She tells spontaneously that she feels like being five years old. Support of the tense muscles in the back intensifies the crying, but only in a good way. After a brief explanation, the patient seems to understand the connection between the feelings she holds back and the tensions in her back causing the problems. After the session she is free of pain and happy. She is told to exercise with: "Cry baby! And do not let them stop you because you need to cry." Exercise for the mother: Do not stop her crying. Just hold her and allow her to feel whatever she feels.*
>
> *At the second session the mother tells that the child cried at home, even a lot! And that was a relief! Her back has improved considerably with episodes of pain only twice the last month compared to normally twice a week.*

This is a typical example and it is estimated that one child in five suffers from chronic pain in Denmark. The pain is not due to any organic damage. Holding gives immediate relief. Teaching holding to the mother reduces almost immediately the back pain by a factor of four. The combination of teaching the child and the parent, and demonstrating the process of holding and healing to both of them, is very powerful. Later in the course, the father is also taught how to give holding. Both parents were unable to give holding, hence the problem of the child.

## CONCLUSIONS

The child needs holding more than anything, because his own powers are too weak to realize its purpose without help. When the holding fails, the child adapts by modifying his or her own existence through taking or making decisions that transforms it into the different person the parents can see, acknowledge, accept, respect, and care for. This is a highly intelligent mechanism for adaptation and survival. Unfortunately the price is that the child loses talent, power, and psychosexual potency resulting in poor quality of life, poor health, and low ability to function.

Fortunately, the damage that the child does to itself is reversible. It is possible, and not so difficult, to take the child into the simplest kind of existential therapy and help it heal spontaneously by giving it adequate holding: awareness of his learning and curious mind, respect for his vulnerable feelings, care for his new and innocent life, acceptance of his precious body, and acknowledgment of his magnificent and gifted soul. A much more effective and lasting strategy than to give the child holding yourself is, of course, to educate his parents to give the holding needed. The essence of the problem is often that the parents do not take responsibility and as this is often a very sensitive issue; timing is everything when it comes to educating the parents. Sometimes this is not possible, and a negative reaction from the individual feeling of guilt in the obviously insufficient parent must be avoided, as this often makes the situation more difficult for the child. In some of these extreme cases, the child has to be removed from his family in order to prevent further damage.

When a child with a poor quality of life grows up it will normally turn into an adult with a poor quality of life. If this adult gets the holding he or she needed as a child, in the proper therapeutic setting, the old wounds will heal[4]. So it is never too late to break the chain of misfortune running in so many families. Quality of life is our birthright, and if we cannot get a good life as children, we can at least get it as adults. But many problems of our children can be helped today by small means. Education of the parents in holding seems mandatory when the child is not thriving. Often a few words, in an appropriate moment, can make an important difference.

## ACKNOWLEDGMENTS

This study was supported by grants from The 1991 Pharmacy Foundation, as well as by supplementary grants from Goodwill-fonden, the JL-Foundation, E. Danielsen and Wife's Foundation, Emmerick Meyer's Trust, the Frimodt-Heineken Foundation, the Hede Nielsen Family Foundation, Petrus Andersens Fond, Wholesaler C.P. Frederiksens Study Trust, Else & Mogens Wedell-Wedellsborg's Foundation and IMK Almene Fond. The research was approved by the Copenhagen Scientific Ethical Committee under number (KF)V.100.2123/91.

## BIOSKETCHES

**Søren Ventegodt, MD,** is the Director of the Quality of Life Research Center in Copenhagen, Denmark. He is also responsible for a Research Clinic for Holistic Medicine in Copenhagen and is a popular speaker throughout Scandinavia. He has published numerous scientific or popular articles and a number of books on holistic medicine, quality of life, and quality of working life. His most important scientific contributions are the comprehensive SEQOL questionnaire, the very short QoL5 questionnaire, the integrated QOL theory, the holistic process theory, the life mission theory, and the Danish Quality of Life Research Survey, 1991–94 in cooperation with the University Hospital of Copenhagen and the late pediatric professor Bengt Zachau-Christiansen. E-mail: ventegodt@livskvalitet.org. Website: www.livskvalitet.org/

**Joav Merrick, MD, DMSc,** is Professor of Child Health and Human Development affiliated with the Zusman Child Development Center, Division of Pediatrics and Community Health at the Ben Gurion University, Beer-Sheva, Israel; the Medical Director of the Division for Mental Retardation, Ministry of Social Affairs, Jerusalem; and the Founder and Director of the National Institute of Child Health and Human Development. He has numerous publications in the field of child and human development, rehabilitation, intellectual disability, disability, health, welfare, abuse, advocacy and prevention. Dr. Merrick received the Peter Sabroe Child Award for outstanding work on behalf of Danish Children in 1985 and the International LEGO-Prize ("The Children's Nobel Prize") for an extraordinary contribution towards improvement in child welfare and well being in 1987. E-mail: jmerrick@internet-zahav.net. Website: www.nichd-israel.com
